# PKCζ and JNK signaling regulate radiation-induced compensatory proliferation in parotid salivary glands

**DOI:** 10.1371/journal.pone.0219572

**Published:** 2019-07-09

**Authors:** Wen Yu Wong, Sydney Allie, Kirsten H. Limesand

**Affiliations:** 1 Cancer Biology Graduate Interdisciplinary Program, University of Arizona, Tucson, Arizona, United States of America; 2 Department of Nutritional Sciences, University of Arizona, Tucson, Arizona, United States of America; University of Kentucky, UNITED STATES

## Abstract

Radiotherapy is a common treatment option for head and neck cancer patients; however, the surrounding healthy salivary glands are often incidentally irradiated during the process. As a result, patients often experience persistent xerostomia and hyposalivation, which deceases their quality of life. Clinically, there is currently no standard of care available to restore salivary function. Repair of epithelial wounds involves cellular proliferation and establishment of polarity in order to regenerate the tissue. This process is partially mediated by protein kinase C zeta (PKCζ), an apical polarity regulator; however, its role following radiation damage is not completely understood. Using an *in vivo* radiation model, we show a significant decrease in active PKCζ in irradiated murine parotid glands, which correlates with increased proliferation that is sustained through 30 days post-irradiation. Additionally, salivary glands in PKCζ null mice show increased basal proliferation which radiation treatment did not further potentiate. Radiation damage also activates Jun N-terminal kinase (JNK), a proliferation-inducing mitogen-activated protein kinase normally inhibited by PKCζ. In both a PKCζ null mouse model and in primary salivary gland cell cultures treated with a PKCζ inhibitor, there was increased JNK activity and production of downstream proliferative transcripts. Collectively, these findings provide a potential molecular link by which PKCζ suppression following radiation damage promotes JNK activation and radiation-induced compensatory proliferation in the salivary gland.

## Introduction

While radiotherapy is an effective treatment strategy for head and neck cancer, an unfortunate side effect is damage to the surrounding healthy salivary glands. This damage often leads to persistent xerostomia, which exacerbates other oral complications and decreases the patient’s quality of life. Although various therapeutic modalities exist [1–4] to combat this phenomenon, there is currently no definitive cure available for radiation-induced hyposalivation. Therefore, a comprehensive understanding of the molecular components that regulate cellular function in response to radiation is crucial to develop strategies to mitigate salivary gland damage.

In most tissue types, cell death during injury leads to the release of mitogenic factors that stimulate compensatory proliferation, a process by which mitotic division is induced to replace the cells lost. The compensatory proliferation response has been demonstrated in imaginal wing discs of *Drosophila melanogaster* [5–8], murine keratinocytes [9,10], and livers of injured mice [11–13]. Similar to these models, salivary glands undergo increased proliferation following radiation-induced damage as early as five days post-treatment [14–19]. Elevated proliferation is still observed at days 30, 60, and 90 post-radiation suggesting the upstream signaling cues remain present [14,15,18]. Compensatory proliferation has been reported in a heterogeneous population of cells and currently there is considerable debate on which population is required to respond to radiation damage [20,21]. This prolonged compensatory proliferation response following radiation correlates with salivary gland hypofunction, as measured by decreased stimulated salivary flow rate and differentiation state (e.g. amylase enzyme production) [18,22,23]. Interestingly, upon administration of therapeutic agents that restore salivary secretion in irradiated mice, proliferation decreases and differentiation increases to levels similar to untreated mice [18,19,24,25]. This suggests that initial stimulation of compensatory proliferation may be necessary to recoup cellular loss; however, a sustained proliferative response prevents further downstream regenerative reprogramming such as differentiation, reepithelization, and tissue remodeling that are necessary for organ function. A better understanding of the regulation of compensatory proliferation following radiation damage in salivary glands thus stands to provide initial insights into why organ function is not restored.

Following tissue injury, the reestablishment of adhesion and polarity is thought to generally limit proliferation through contact inhibition. Protein kinase C zeta (PKCζ), a serine/threonine kinase that is a part of the Par3/Par6/PKC complex, promotes the establishment of apical-basalolateral polarity in a number of exocrine tissues, including the salivary glands [19,26,27]. Loss of Par3/Par6/PKC complex function is known to promote hyperproliferation, development of carcinomas, and prevention of tissue regeneration [19,28–31]. Importantly, previous research has shown that PKCζ is required for the restoration of salivary gland function following radiation damage [19]. PKCζ^-/-^ mice were unable to restore salivary flow rates despite administration of therapeutics known to restore salivary function. While this study highlights the necessity of PKCζ during the regenerative process, the mechanistic link between PKCζ and proliferation following radiation damage is poorly understood.

Previous studies have implicated Jun N-terminal kinase (JNK), a mitogen activated protein kinase (MAPK), as a key promoter of compensatory proliferation. JNK responds to extracellular stressors and activates proliferation-inducing transcription factors such as c-Jun, ATF, and Elk1 [32,33]. Notably, contradicting functions have been described regarding the roles of PKCζ and MAPKs in response to injury. For example, in human bronchial epithelial cells, stimulation of PKCζ activates ERK and JNK, resulting in extracellular matrix degradation and increased cellular invasion [34]. In contrast, a PKCζ deficient lung cancer mouse model showed increased interleukin-6 production that lead to increased proliferation and tumorigenesis [35]. PKCζ can functionally display diverse properties depending on the cellular context and type of injury. Thus, determining how these pathways respond following radiation damage within the salivary glands could provide important insights into the defective regenerative mechanism underlying salivary hypofunction.

## Material and methods

### Mice and radiation treatment

Experiments in this study were conducted in female FVB mice and both male and female C57BL/6J and *Prkcz* ^-/-^ mice. For each experiment, at least four animals were used per treatment group. Mice were maintained and treated in agreement with protocols approved by the University of Arizona Institutional Animal Care and Use Committee (IACUC). *Prkcz* ^-/-^ mice were generated and maintained as previously described [18,19]. One dose of 5 Grey (5Gy) was administered with a ^60^Cobalt Teletherapy Instrument from Atomic Energy of Canada Ltd Theratron-80. The head and neck region of the mice were exposed while the rest of the body was shielded from radiation with >6mm thick lead to avoid systemic effects. Mice were anesthetized with an intramuscular injection of ketamine/xylazine (50mg/kg:10mg/mL) before radiation treatment and were monitored until they regained consciousness. Radiation dosage calculations and maintenance of the cobalt source are conducted by the Experimental Radiation Shared Service of the Arizona Cancer Center.

### Immunoblotting

Whole protein lysates from parotid glands of FVB, wild type C57BL/6J and *Prkcz* ^-/-^ mice were harvested and processed for immunoblotting as previous described [19,36]. Primary cell lysates were processed in the same fashion. Briefly, samples were lysed in RIPA buffer with 5mM sodium orthovanadate (Fisher Scientific, Hampton, NH), protease inhibitor cocktail (Sigma-Aldrich, St. Louis, MO) and 100mM PMSF (Thermo Scientific, Waltham, MA). The Coomassie Plus-The Better Bradford Assay (Thermo) was used to determine protein concentrations and 30–100μg of total lysate was used. The following antibodies were used: anti-PARD3 (Abcam), anti-PARD6 (Proteintech), anti-total PKCζ (Cell Signaling), anti-pPKCζ (T560) (Abcam), anti-phospho-c-Jun (S63) (Cell Signaling), and anti-beta-tubulin (Thermo Scientific). Restore Western Blotting Stripping Buffer (Fisher) was used to strip membranes and reprobed for loading controls.

### Immunofluorescent staining

Salivary glands were dissected at predetermined time points for formalin-fixed paraffin-embedded (FFPE) samples as previously described [19,36]. Briefly, samples were fixed in formalin and cut to 4μm thickness by IDEXX BioResearch (Columbia, MO). Slides were rehydrated in graded ethanol, permeabilized in 0.02% Triton X-100, and antigen retrieval in 1mM citric acid buffer (pH 6.8). The slides were then blocked in 0.5% NEN (PerkinElmer, Waltham MA) and incubated in primary antibody overnight at 4°C. Secondary antibodies were added for 1 hour at room temperature with Alexa Fluor 594 or Alexa Fluor 488 (Thermo Scientific). Samples were counterstained with DAPI (1μg/mL) and mounted with ProLong^™^ Gold Antifade Reagent (Life Technologies). Fluorescently stained slides were stored at 4°C for no longer than 5 days until imaging. The following antibodies were used: anti-Ki67 (Cell Signaling) and anti-pPKCζ (T560) (Abcam). Images were taken with a Leica DM5500 microscope (Leica Microsystems, Wetzlar, Germany) and a Spot Pursuit 4 Megapixel CCD camera (Diagnostic Instruments, Sterling Heights, MI). Images were processed with ImagePro 6.3 (Media Cybernetics, Silver Spring MD) and ImageJ (NIH). Analysis of Ki67-positive cells was performed manually by counting positive cells from at least 7 images per slide per treatment condition. A minimum of three mice per group was analyzed. Percentages of total Ki67 positive cells in the acinar compartment was divided by the total number of cells in this compartment. During analysis, the ductal compartment was designated based on morphological features as previously described, such as rounded structures, the presence of a lumen, and tight cell-cell contact [21]. The ductal compartment includes the excretory and striated ducts, as well as some intercalated ducts. Thus, the acinar compartment comprises all the remaining cell types in the salivary epithelium: mainly acinar and myoepithelial cells, as well as some of the intercalated ducts that based on morphology could not be identifiable as ducts. Analysis of pPKCζ (T560) area was quantified as previously described [18]. Briefly, slides were imaged using the same fluorescent parameters. Morphometric analysis was performed with ImagePro 7.0 software (Media Cybernetics, Silver Spring, MD). Positive area was determined from at least 10 fields of view (FOV = 0.39mm^2^) with a coefficient of variation <7.5% which did not improve with greater numbers of observations per section. Data are expressed as percentage of pPKCζ (T560) intensity area over total area and the threshold fluorescent range (5x greater than background) was equivalent for all slides imaged.

### JNK kinase assay

JNK kinase activity was detected using a JNK activity assay kit according to the manufacturer’s protocol (RayBiotech, Norcross, GA). Briefly, JNK kinase was immunoprecipitated from sample lysates using a JNK-specific antibody. The activity of JNK was then determined using recombinant c-Jun as the substrate. Phosphorylation of c-Jun was detected using immunoblotting techniques. The BioTek Gen5 (BioTek Instruments, Winooski, Vermont) was used for readings of protein concentrations.

### Primary cell culture

Parotid glands were removed from euthanized mice and cultured as primary cells as previously described [36,37]. Briefly, the glands were minced in dispersion media, mechanically agitated, cultured in primary cell culture media, and grown on rat tail collagen plates (Corning, Corning, NY). On day 1 after dissection, cells were exposed to a single dose of 5Gy radiation. For the PKCζ inhibitor experiments, the cells were grown to sub-confluency and treated with either 20 μM PKCζ pseudosubstrate inhibitor (PPI) (Calbiochem) or vehicle for 2 hours. After 2 hours, JNK kinase activity, c-Jun phosphorylation, and downstream proliferative promoting transcripts were subsequently measured. For the JNK inhibitor experiments, cells were cultured with 10 μM SP600125 (JNK inhibitor) or DMSO vehicle control on day 4 following radiation treatment. Cells were harvested on day 5 and protein lysates or RNA were collected for downstream analyses as described.

### Real-time RT-PCR

Parotid glands were removed from mice, stored in RNALater Stabilization Reagent (Qiagen, Valencia, CA), and processed as previously described [19,36]. Briefly, samples were isolated with the RNeasy Mini Kit (Qiagen) and reversed transcribed with SuperScript IV Reverse Transcriptase (Invitrogen). Samples were analyzed in triplicate for each cDNA sample (3–5 mice per condition and at least 3 independent primary cell preps) with an iQ5 Real-Time PCR Detection System (Biorad). The data was analyzed using the 2^-ΔΔCT^ method [38]. Results were normalized to GAPDH, which remains unchanged in response to treatment. Normalized values were graphed as relative fold-change compared to controls. The following primers were purchased from Integrated DNA Technologies (Coralville, IA): GAPDH (FWD: 5’-ACC ACA GTC CAT GCC ATC AC-3’; REV: 5’-CAC CAC CCT GTT GCT GTA GCC-3’); CCND1 (FWD: 5’-GCG TAC CCT GAC ACC AAT CTC-3’; REV: 5’-CTC CTC TTC GCA CTT CTG CTC-3’); PDE3A (FWD: 5’-CCT GGA CTA GCG TGC TTA GGA-3’; REV: 5’-CAG GCG ACC TTG AAC CTC T-3’); NFATC2 (FWD: 5’-TCA TCC AAC AAC AGA CTG CCC-3’; REV: 5’-GGG AGG GAG GTC CTG AAA ACT-3’), MT1F (FWD: 5’-ACT TTC CCT TAT CCC ATC CAC C-3’; REV: 5’-TGA GAT CCA GAG TTG TCG TAC A-3’).

### Statistics

Data were analyzed using Prism 6.04 (GraphPad, La Jolla, CA). All values are reported as the mean ± standard error of at least three independent experiments. Student’s t-test was applied to results in which only two groups (untreated vs. inhibitor or untreated vs. day 5 irradiated) were compared. A one-way analysis of variance (ANOVA) test and a Tukey-Kramer test for multiple comparison was used to compare results within different group means and was considered significantly different at p<0.05.

## Results

### Radiation decreases pPKCζ but not total levels of the PKCζ/Par3/Par6 complex

**Fig 1:**
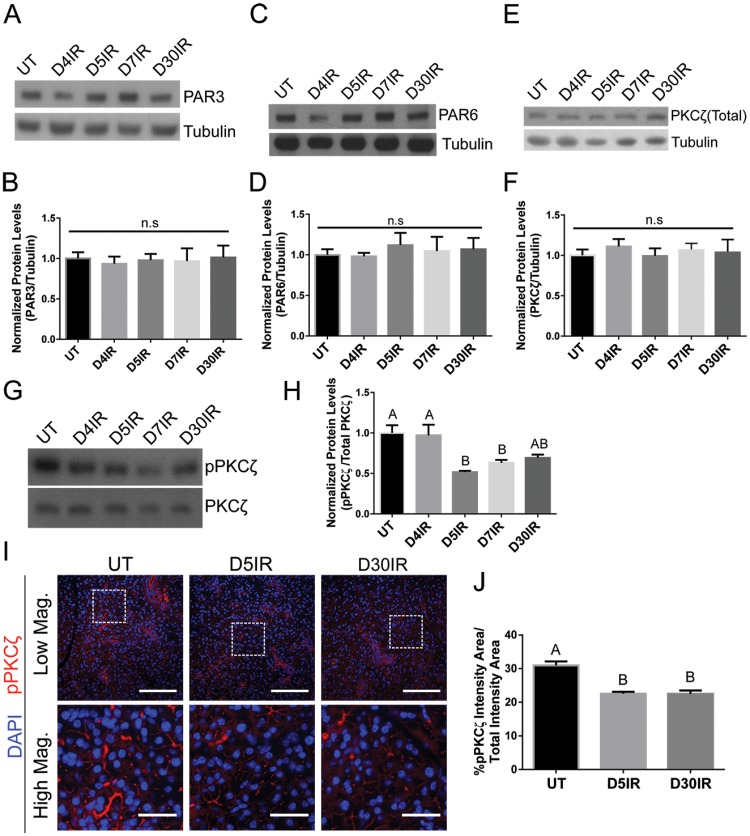
Radiation decreases pPKCζ-T560 but not total levels of the PKCζ/Par3/Par6 complex. FVB mice were either untreated (UT) or irradiated (IR) with 5Gy and dissected on days 4, 5, 7, and 30 following radiation treatment. Total protein levels of (A-B) Par3, (C-D) Par6, (E-F), total PKCζ, and (G-H) pPKCζ-T560 were evaluated following radiation treatment. Immunoblots were re-probed with β-tubulin or PKCζ as a loading control. (I) Immunofluorescent staining was used to determine the intensity area of pPKCζ-T560 (red) in comparison to the total area. Composite images with DAPI (blue) are presented in both high and low magnification views (scale bar for high magnification = 30 μm, low magnification = 100 μm). (J) Quantification of the percentage of pPKCζ-T560 positive area within the parotid gland. Results are presented from at least three mice per condition; I-J used 10–15 images per mouse; error bars denote mean ± SEM. Significant difference (p<0.05) was determined by a Tukey-Kramer test for multiple comparisons. Treatment groups with different letters above the bar graphs are significantly different from each other.

PKCζ has been previously shown to be essential in regulating salivary progenitor cell proliferation [19] following radiotherapy, but little is known about its role in the remaining salivary cells following damage. Thus, to investigate the possible role of the PKCζ/Par3/Par6 complex following radiation treatment, immunoblotting was performed to determine total protein levels at days 4, 5, 7, and 30 following radiation treatment. Total levels of PKCζ, Par3, and Par6 did not change with radiation treatment in comparison to untreated (Fig 1A–1F). PKCζ is active when it is phosphorylated in the activation loop at threonine 410 (T410) leading to autophosphorylation at T560. Thus, to determine whether PKCζ is active following radiation treatment, levels of phosphorylated-PKCζ at T560 (pPKCζ-T560) were evaluated, and a decrease in pPKCζ is observed at days 5, 7, and 30 following radiation treatment (Fig 1G–1H). Immunofluorescent staining for pPKCζ-T560 was performed to compare fluorescent intensity area to total tissue area (Fig 1I and 1J). In untreated mice, pPKCζ is localized to the apical region of cells and displays a higher percentage of pPKCζ-positive cell area. Comparatively, irradiated mice at days 5 and 30 display lower percentage of pPKCζ-positive cell area which correlates with the time points at which pPKCζ was decreased in the immunoblotting analysis. These results suggest that radiation disrupts pPKCζ levels as early as day 5 and these levels decreased chronically to day 30.

### Depletion of PKCζ induces proliferation *in vivo*

**Fig 2:**
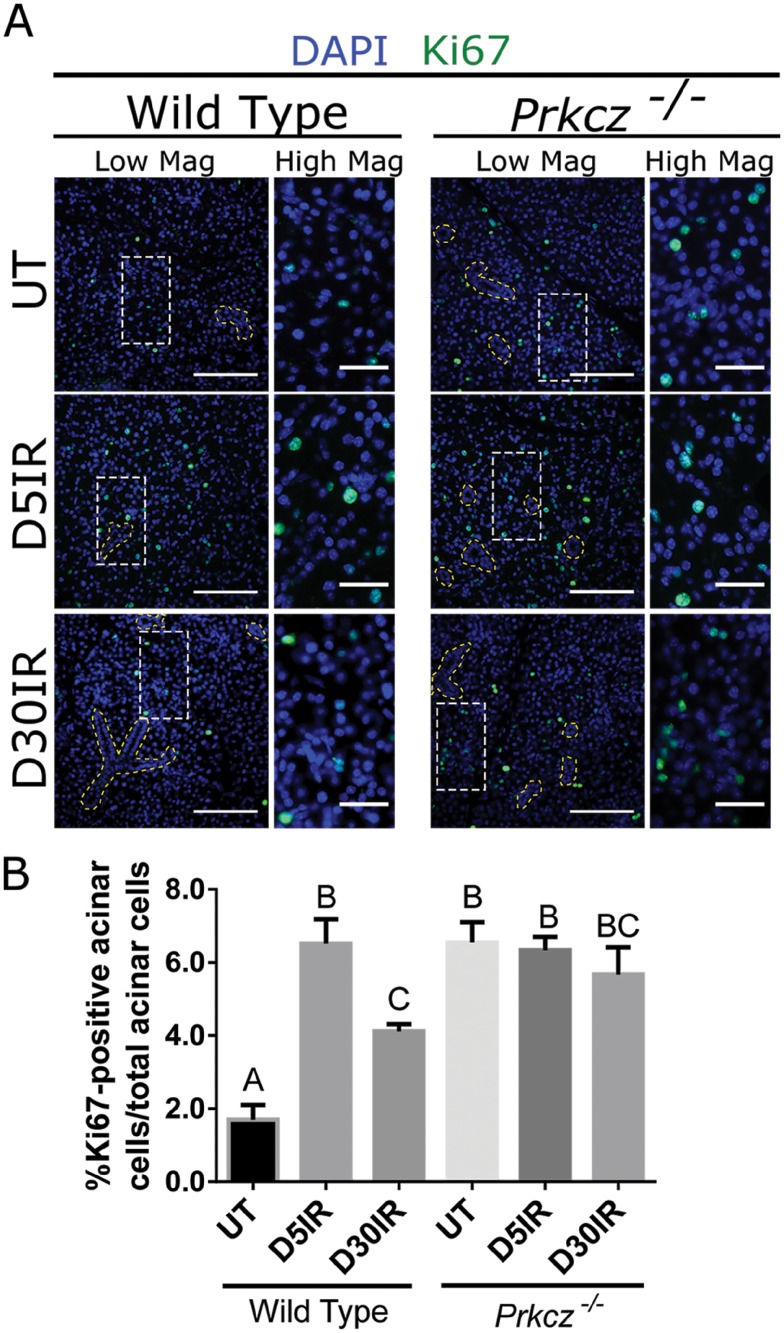
Depletion of PKCζ induces proliferation *in vivo*. Wild Type C57BL/6J and *Prkcz* ^-/-^ mice were either untreated (UT) or irradiated (IR) with 5Gy and dissected on days 5 and 30 following radiation treatment. (A) Immunofluorescent staining with Ki67 (green) was used to determine the number of proliferating cells in comparison to the total number of cells in the acinar compartment. Composite images with DAPI (blue) are presented in both high and low magnification views (scale bar for high magnification = 30 μm, low magnification = 100 μm). The yellow dotted outline represents the ductal compartment while the rest of the glandular area represents the acinar compartment. (B) Quantification of A. Results are presented from at least four mice per condition; A-B used 10–15 images per mouse; error bars denote mean ± SEM. Significant difference (p<0.05) was determined by a Tukey-Kramer test for multiple comparisons. Treatment groups with different letters above the bar graphs are significantly different from each other.

Studies in *Drosophila* and murine keratinocytes suggest a relationship between loss of cell polarity and loss of proliferation control [7,9,39]. Because radiation decreases the polarity regulator, pPKCζ, the effect of radiation on proliferation was evaluated. Tissues from untreated and irradiated wildtype C67BL/5J mice were evaluated for the proliferation marker Ki67 by immunofluorescent staining (Fig 2A and 2B). Because decreased pPKCζ spanned from days 5 to 30 (Fig 1), these time points were chosen for further proliferation evaluation. In untreated mice, the percentage of Ki67 positive cells in the acinar compartment is 1.8%. In comparison, when the wildtype mice received one treatment of 5Gy radiation, the percentage of Ki67 positive cells increases significantly to 6.5% and 3.7% at days 5 and 30 post-radiation, respectively. To test whether the elevated proliferative response can be mediated by PKC, mice with a genetic disruption in PKCζ (*Prkcz* ^-/-^) were either untreated or irradiated with 5Gy treatment and Ki67 immunofluorescent staining was used to detect proliferating cells. In untreated *Prkcz* ^-/-^ mice, 5.5% of cells stained Ki67 positive which is a similar level as irradiated wildtype mice at day 5. Radiation did not further elevate the percentage of Ki67 positive cells in *Prkcz* ^-/-^ mice at days 5 (6.3%) and day 30 (5.6%). These data suggest that radiation induces a compensatory proliferation response that is sustained to day 30, and this compensatory proliferation response could be regulated by PKCζ.

### Radiation induces JNK signaling *in vivo*

**Fig 3:**
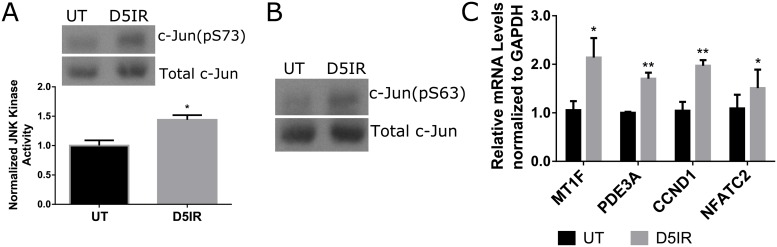
Radiation induces JNK signaling in vivo. FVB mice were either untreated (UT) or irradiated (IR) with 5Gy and dissected on day 5 following radiation treatment to evaluate JNK signaling. (A) Relative JNK kinase activity was measured by incubating immunoprecipitated JNK in the presence of ATP and a c-Jun substrate. The amount of phosphorylated c-Jun (S73) was detected via immunoblots. (B) Levels of phosphorylated c-Jun (S63) were determined following radiation treatment. Total c-Jun was probed as a loading control. (C) Relative mRNA levels of MT1F, PDE3A, NFATC2, and CCND1 were determined by RT-PCR and normalized to GAPDH. Results are presented from at least four mice per condition. Significant difference (p<0.05) was determined by Student’s t-test. *(p<0.05), **(p<0.01).

Since alterations in PKCζ have been linked to JNK signaling [33,35], we investigated whether JNK signaling was affected following radiation treatment. The onset of radiation-induced compensatory proliferation (Fig 2) and the decrease in pPKCζ (Fig 1) occurs on day 5 post-irradiation; therefore, this time point chosen for further evaluation. JNK kinase activity, c-Jun phosphorylation, and downstream proliferative mRNA transcripts (MT1F, NFATC2, PDE3A, and CCND1) [13,32,33,40] were evaluated in untreated and irradiated mice. There are higher levels of JNK activity in salivary tissue samples collected on day 5 post-irradiation compared to untreated tissues (Fig 3A), which correlates with an increase in phosphorylated c-Jun at serine 63 (S63) (Fig 3B). To evaluate whether JNK downstream signaling also occurs, RT-PCR was performed to determine whether JNK-mediated proliferation-promoting transcripts such as MT1F, NFATC2, PDE3A, and CCND1 were altered in salivary tissues following radiation treatment. In comparison to untreated mice, irradiated mice display significantly elevated mRNA transcripts for MT1F, NFATC2, PDE3A, and CCND1 (Fig 3C). These results suggest that radiation upregulates the JNK signaling axis.

### Inhibition of JNK signaling with SP600125 in irradiated cells

**Fig 4:**
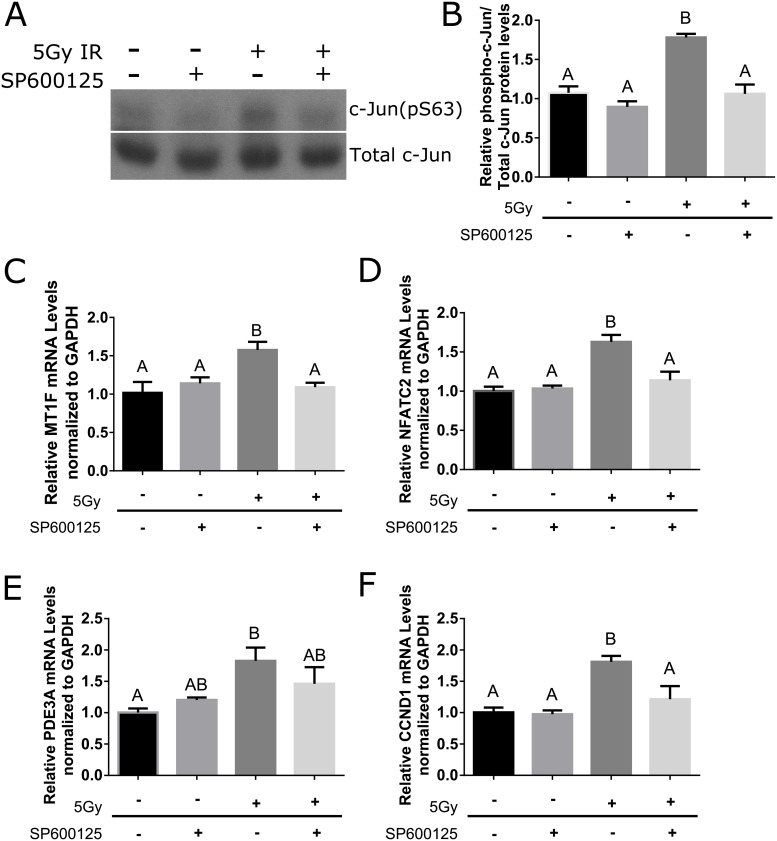
Inhibition of JNK signaling with SP600125 in irradiated cells. Parotid salivary glands from FVB mice were dissected and cultured as primary cell cultures. One day after dissection, the primary cells were irradiated with 5Gy and cell lysates or RNA were collected on Day 5 after radiation treatment. On Day 4 after radiation treatment, the cells were either treated with 10 μM SP600125 or DMSO vehicle control. (A) Effects of SP600125 treatment on phosphorylated c-Jun (S63) in primary salivary cells were evaluated by immunoblotting. Blots were reprobed for total levels of c-Jun as a loading control. (B) Quantification by densitometry of A normalized to DMSO vehicle control. (C-F) Relative MT1F, PDE3A, NFATC2, and CCND1 mRNA levels determined by RT-PCR and normalized to GAPDH. Results are presented from at least three independent primary cell preparations; error bars denote mean ± SEM. Significant difference (<0.05) was determined by a Tukey-Kramer test for multiple comparisons. Treatment groups with different letters above the bar graphs are significantly different from each other.

To confirm that MT1F, PDE3A, NFATC2, and CCND1 where regulated by JNK signaling, untreated and irradiated primary cell cultures were treated with 10 μM of the specific JNK inhibitor, SP600125. Since JNK activity was observed at day 5 post-radiation, treatment with 10 μM SP600125 was started on day 4 post-radiation. As a surrogate readout for JNK inhibition, levels of a JNK substrate, phosphorylate c-Jun was tested. Phosphorylated c-Jun (S63) is reduced in cells treated with SP600125 (Fig 4A and 4B). To determine whether inhibition of JNK would result in a decrease of proliferative transcripts, RT-PCR was used. Cells treated with 10 μM SP600125 show lower mRNA expression of MT1F, PDE3A, NFATC2, and CCND1 in comparison to irradiated cells (Fig 4C–4F). These data suggest that inhibition of JNK activity in irradiated cells can decrease proliferative regulators such as MT1F, PDE3A, NFATC2, and CCND1.

### Modulation of PKCζ increases JNK signaling

**Fig 5:**
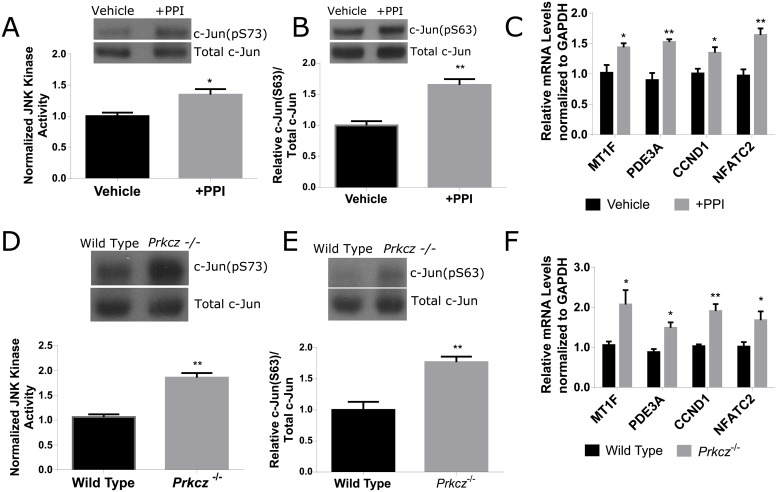
Modulation of PKCζ increases JNK signaling. Parotid salivary glands from FVB mice were dissected and cultured as primary cell cultures. At sub-confluency, the cells were treated with 20 μM PKCζ pseudosubstrate inhibitor (PPI) or vehicle control. (A) Relative JNK kinase activity was measured by incubating immunoprecipitated JNK in the presence of ATP and c-Jun substrate in cells treated with 20 μM PPI or vehicle control. The amount of phosphorylated c-Jun (S73) was detected via immunoblots. (B) Total protein levels for phosphorylated c-Jun (S63) was determined following PPI inhibition. Immunoblots were reprobed with total c-Jun as a loading control. Relative quantification is depicted below the blot. (C) Relative mRNA levels of MT1F, PDE3A, NFATC2, and CCND1 were determined by RT-PCR and normalized to GAPDH as a loading control. Lysates and mRNA were collected from C57BL/6J wildtype and *Prkcz* ^-/-^ mice. (D) Relative JNK kinase activity was measured by incubating immunoprecipitated JNK in the presence of ATP and c-Jun substrate in wildtype or *Prkcz* ^-/-^ mice. The amount of phosphorylated c-Jun (S73) was detected via immunoblots. (E) Total protein levels for phosphorylated c-Jun (S63) was determined in wildtype or *Prkcz* ^-/-^ mice. Immunoblots were reprobed with total c-Jun as a loading control. Relative quantification is depicted below the blot. (F) Relative mRNA levels of MT1F, PDE3A, NFATC2, and CCND1 were determined by RT-PCR and normalized to GAPDH as a loading control. Results are presented from at least four mice for *in vivo* experiments or three independent *in vitro* primary cell culture experiments per condition; error bars denote mean ± SEM. Significant difference (p<0.05) was determined by Student’s t-test. *(p<0.05), **(p<0.01).

After observing a decrease in pPKCζ with a corresponding increase in JNK signaling following radiation damage, we hypothesized that PKCζ can regulate JNK activity and signaling. To study the role of PKCζ, we used an *in vitro* primary cell culture model treated with a specific PKCζ pseudosubstrate inhibitor (PPI). Primary cells were grown to sub-confluency and were treated with 20 μM PPI or vehicle. Cells treated with the PKCζ inhibitor show elevated JNK activity (Fig 5A) and c-Jun phosphorylation (Fig 5B) in comparison to vehicle control. In conjunction, cells treated with the PKCζ inhibitor express elevated mRNA levels of MT1F, PDE3A, NFATC2, and CCND1 (Fig 5C). To further determine whether JNK signaling is regulated by PKCζ, JNK activity and downstream proliferative genes were analyzed in mice depleted of PKCζ. Similar to *in vitro* experiments, salivary glands from *Prkcz* ^-/-^ mice show elevated JNK activity (Fig 5D), elevated c-Jun phosphorylation (Fig 5E) and elevated proliferative mRNA expression (Fig 5F) in comparison to wildtype C67BL/5J mice. These data suggest that modulation of PKCζ can regulate downstream JNK signaling.

## Discussion

Tissue repair and regeneration after injury requires multiple processes including the integration of polarity, which is the positional identity cue of a cell, with preexisting structures. The establishment of epithelial polarity is necessary to repress proliferation and promote differentiation during the wound healing process. However, the mechanistic contribution of apical polarity regulators following radiation damage has not been fully elucidated. Here, we demonstrate that a cell polarity regulator, PKCζ and downstream JNK signaling, mediate radiation-induced compensatory proliferation in murine parotid glands.

JNK has been implicated in compensatory proliferation, regeneration, as well as, apoptosis [13,32,33]. The dual roles of JNK signaling as a mediator of apoptosis or as a mediator of cell proliferation raises the question of how one of these downstream outcomes become favored in a temporal manner following radiation damage. Some insights to this question can be gleaned from previous work on the apoptotic response of irradiated salivary glands, whereby apoptosis can be detected as early as four hours after radiation treatment, peaks at 24 hours, and returns to basal levels by 72 hours [41–44]. In contrast, the current study demonstrates that radiation-induced JNK signaling is observed at day 5 post-radiation, a time point beyond the apoptotic response and the beginning of the compensatory proliferation response. Although the exact mechanism controlling the balance between apoptosis and proliferation is unclear, the identification that PKCζ can modulate JNK signaling sheds some light on this process since radiation induces the decrease in pPKCζ (T560) at day 5 post-irradiation. Given that PKCζ is essential for tissue regeneration and re-establishment of salivary function, development of therapeutic strategies to increase active PKCζ serves as a promising approach to combat harmful side effects of radiotherapy in patients with head and neck cancer.

The upregulation of JNK signaling observed in this study parallels a study where submandibular glands from rats irradiated with 20Gy displayed elevated pJNK on day 7 in comparison to untreated mice [45]; however, the authors did not evaluate potential upstream regulators. Here, we demonstrate that PKCζ could regulate JNK signaling and proliferative transcripts utilizing an *in vitro* PKCζ pseudosubstrate inhibitor (PPI) or *Prkcz*^-/-^ mice. While activation of JNK promotes intestinal stem cells to proliferate and replenish damaged cells [46], aberrantly high or prolonged JNK signaling results in accumulation of mis-differentiated cells and neoplastic transformation involving excess proliferation [47]. Perhaps, radiation-induced JNK signaling is preventing salivary restoration through the disruption of the differentiation process in a similar manner as intestinal cells. Others have suggested that disruption of polarity can activate JNK signaling through a Rho-associated coiled-coil kinase (ROCK)-dependent axis [39], while disruption of Scribble, another polarity regulator, eliminates aberrant cells via upregulation of JNK-regulated endocytosis [48]. It was previously shown that radiation damage can upregulate ROCK signaling [36], which suggests that activation of ROCK signaling following PKCζ disruption may provide additional signals that lead to compensatory proliferation in a positive feedback loop. This suggests the possibility of multiple signaling axes that result in compensatory proliferation and whether the ultimate outcome is beneficial (restoring cell numbers) or detrimental (neoplasia, loss of differentiation) depends on cellular context.

Studies in Drosophila suggest a relationship between disrupted polarity (such as PKC and Scribble) and the promotion of uncontrolled proliferation [7,39], while restoration of polarity helped reestablish tissue integrity [49–51]. Here, we have identified that radiation reduces the levels of pPKCζ without altering total levels of components within the apical complex (Fig 1). The decrease in pPKCζ correlates with an increase in radiation-induced proliferation that is maintained to day 30 (Fig 2), suggesting a continual loss of apical polarity may provide cues that the wound healing process is incomplete. In addition, influences from the surrounding parenchyma, might also promote the radiation-induced proliferation response. Genetic ablation of *Prkcz* ^-/-^ in mice results in elevated proliferation regardless of radiation treatment similar to radiation-induced compensatory proliferation in wildtype control mice (Fig 2: comparison between Wild Type D5IR and *Prkcz* ^-/-^ UT). This strongly suggests that proper regulation of PKCζ is necessary to repress radiation-induced compensatory proliferation. Future research into understanding why and how decreased pPKCζ persists to day 30 could provide insights into why the salivary glands fail to restore function following radiation treatment.

While compensatory proliferation is an evolutionarily conserved mechanism that is critical in repopulating damaged tissue, improper signaling can inadvertently stimulate excessive proliferation and loss of differentiation [12,52–55]. Further understanding of how the epithelial cells and parenchyma respond to the context-specific, spatiotemporal integration of signaling inputs and outputs upon radiation damage could provide a better understanding of how to regulate compensatory proliferation.
